# Development and Validation of a Radiomics Nomogram for Differentiating Mycoplasma Pneumonia and Bacterial Pneumonia

**DOI:** 10.3390/diagnostics11081330

**Published:** 2021-07-24

**Authors:** Honglin Li, Ting Li, Qinxin Cai, Xiaozhuan Wang, Yuting Liao, Yuanxiong Cheng, Quan Zhou

**Affiliations:** 1 Department of Radiology, The Third Affiliated Hospital of Southern Medical University, Guangzhou 510000, China; lihonglin163@163.com (H.L.); Teddymiao0211@163.com (Q.C.); WXZ24568@163.com (X.W.); 2 Department of Respiratory Medicine, The Third Affiliated Hospital of Southern Medical University, Guangzhou 510000, China; liting_0514@163.com; 3 GE Healthcare, Guangzhou 510000, China; lytting@outlook.com

**Keywords:** mycoplasma pneumonia, bacterial pneumonia, nomogram, multidetector computed tomography

## Abstract

Objectives: To develop and validate a radiological nomogram combining radiological and clinical characteristics for differentiating mycoplasma pneumonia and bacterial pneumonia with similar CT findings. Methods: A total of 100 cases of pneumonia patients receiving chest CT scan were retrospectively analyzed, including 60 patients with mycoplasma pneumonia and 40 patients with bacterial pneumonia. The patients were divided into the train set (*n* = 70) and the test set (*n* = 30). The features were extracted from chest CT images of each patient by AK analysis software, then univarite analysis, spearman correlation analysis, and least absolute shrinkage and selection operator (LASSO) were utilized for dimension reduction in training set. A radiomics model was built by multivariable logistic regression based on the selected features, and a radiomics-clinical multivariable logistic regression model was built by combining imaging radiomics and clinical risk factors (age and temperature). ROC, AUC, sensitivity, specificity, and accuracy were calculated to validate the two models. The nomogram of the radiomics-clinical was built and evaluated by calibration curve. The clinical benefit of the two models was measured by using decision curve. Results: A total of 396 texture features were extracted from each chest CT image, and 10 valuable features were screened out. In the radiomics model, the AUC, sensitivity, specificity, and accuracy for the train set is 0.877, 0.762, 0.821, 78.6%, and for the test set it is 0.810, 0.667, 0.750 and 70.0%, respectively. In the radiomics-clinical model, the AUC, sensitivity, specificity, and accuracy for the train set is 0.905, 0.976, 0.714, 87.1%, and for the test set is is 0.847, 0.889, 0.667 and 80.0%, respectively. Decision curve analysis shows that both the two models increase the clinical benefits of the patients, and the radiomics-clinical model gains higher clinical benefits, compared to the radiomics model. Conclusion: The radiomics-clinical nomogram had good performance in identifying mycoplasma pneumonia and bacterial pneumonias, which would be helpful in clinical decision-making.

## 1. Background

Mycoplasma pneumonia is a major cause of community-acquired pneumonia (CAP) in adults and children, with an epidemic occurring every 3 to 7 years [1]. During epidemics, this microorganism can cause up to 20–40% of CAP in the general population, and up to 70% in closed populations [2]. Pneumonia remains one of the most common causes of death [3], despite significant advances in the worldwide search for anti-bacterial agents to prevent infection in recent years.

The gold standard of mycoplasma pneumoniae diagnosis is the culture method, specifically for pharyngeal throat or tracheal aspiration of pharyngeal swab, pleural puncture fluid or alveolar lavage fluid, mycoplasma pneumoniae culture and separation. Due to the harsh culture conditions of mycoplasma pneumoniae and its slow growth, it lacks the value of early diagnosis. Mycoplasma pneumoniae-DNA testing has certain limitations, as specimen collection may have a higher false negative cost and require certain instruments to complete manual work, heavy workload, and a long time duration [4]. Imaging examination is a commonly used examination method for pneumonia, including X-ray and CT examination. High-resolution computed tomography (HRCT) has unique advantages and is the best imaging examination method for pneumonia patients, but the imaging manifestations of mycoplasma pneumoniae infection have no obvious specificity. Due to the lack of specificity in the imaging manifestations of mycoplasma pneumonia, the differential diagnosis of mycoplasma pneumonia and bacterial pneumonia lacks rapid, accurate, and effective detection methods, so the clinical diagnosis is difficult.

In recent years, the term radiomics has attracted increasing attention. It refers to the process of extracting quantitative features through high throughput and then conducting data analysis to support decisions to transform medical images into high-dimensional and mineable data [5,6]. Advances in pattern recognition tools and in the size of data sets have facilitated the development of radiology, which may improve the predictive accuracy of pathology [7,8]. Radiomics can study multiple imaging features simultaneously and can provide combinations of features that include extracting image features and combining them with other available patient data to enhance decision support models. Radiomics has been successfully applied to the identification, staging, and evaluation of lung cancer [9,10]. Yanling W [11] applied a radiomics nomogram to differentiate pneumonia from acute paraquat lung injury, so it may have the potential to identify pulmonary inflammation.

Thus, our goal was to establish and validate a radiological nomogram that combines radiological characteristics with clinical risk factors to identify mycoplasma pneumonia and bacterial pneumonia, and to provide evidence for early and precise treatment in the clinic.

## 2. Materials and Methods

### 2.1. Patients

This retrospective study was conducted at a single academic medical center and approved by the institutional review board, and the informed consent requirement was waved. This study analyzed the clinical and imaging data of patients diagnosed with mycoplasma pneumonia or bacterial pneumonia in our hospital from January 2018 to December 2019. The inclusion criteria were as follows: (1) patients with mycoplasma pneumonia or bacterial pneumonia; (2) patients had undergone pharyngeal swab or bronchofibroscope alveolar lavage nucleic acid test; (3) chest CT scans are available. The exclusion criteria were as follows: (1) poor image quality; and (2) patients with previous bronchial asthma, chronic obstructive pulmonary disease, kidney or liver disease, recurrent respiratory infections, a history of severe pneumonia but not cured, congenital or secondary immunosuppression or deficiency, connective tissue disease. 

According to the inclusion and exclusion criteria of patients, a total of 100 patients were finally included in this study, including 60 patients with mycoplasma pneumonia and 40 patients with bacterial pneumonia. All 100 patients were randomly divided into a train set (*n* = 70) and a test set (*n* = 30) at a ratio of 7:3 [11]. There were 42 cases of mycoplasma pneumonia in the train set and 28 cases of bacterial pneumonia. In the test set, there were 18 cases of mycoplasma pneumonia and 12 cases of bacterial pneumonia. Clinical data were recorded, including gender, age, body temperature, c-reactive protein, white blood cell count, and neutrophils count.

### 2.2. CT Examinations

A 64-slice spiral CT scanner was used. Scanning parameters: 130 kV, 120 mA, layer thickness 1.0 mm, layer spacing 10 mm, scanning time 2 s, matrix 512 × 512. Hold breath and scan from the apex of the lung to the diaphragm in turn. A high resolution (bone) reconstruction algorithm was used to post-process the image. Region of interest (ROI) was delineated under lung window (Window Width: 1000~2000 Hu, Window Level: −500~−700 Hu).

### 2.3. ROI Delineation

**Figure 1:**
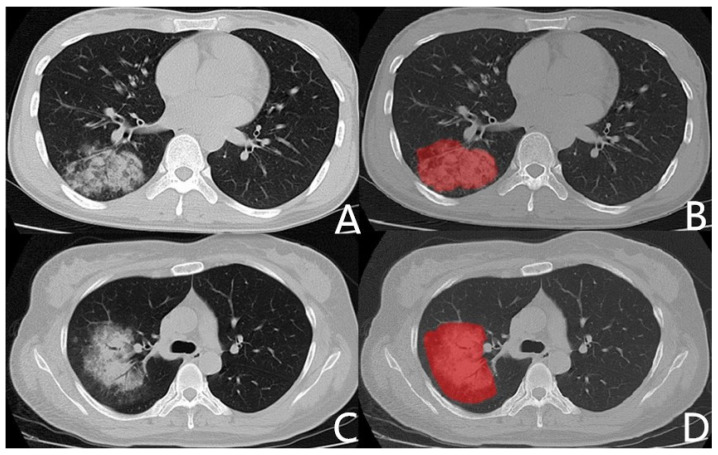
Chest CT images and related ROIs. (**A**): Chest CT of mycoplasma pneumonia. Male, 27 years old, fever for 6 days, maximum temperature 39.8 °C, CRP 73 mg/L, WBC 4.9 × 10^9^/L, neutrophil 67.7%. (**C**): Chest CT of bacterial pneumonia (pseudomonas aeruginosa). Female, 39 years old, fever for 5 days, maximum temperature 37.5 °C, CRP 12 mg/L, WBC 9.8 × 10^9^/L, neutrophil 65.1%. (**B**,**D**): ROI delineated with ITK-SNAP.

All CT images are manually segmented by ITK-SNAP software (Version 2.8; www.itksnap.org, accessed on 15 September 2019). Two radiologists, who had been engaged in chest imaging diagnosis for 5 years and 10 years, respectively, manually delineated ROI on the maximum layer of lesions without knowing the pathological results. The region of interest avoids pleural and pleural effusions. The main, lobe vessels, and bronchi are not included in the ROI. Segment and subsegment bronchus, vessels connected to the lesion are drawn into the ROI, and those who are not connected, are not drawn into the ROI [11]. Figure 1 shows the Chest CT images of patients with mycoplasma pneumonia or bacterial pneumonia and related ROIs.

### 2.4. Features Extraction

CT images and corresponding ROI images were loaded into the AK (Artificial Intelligent Kit, GE Healthcare, Life Science, Guangzhou, China) for feature extraction. A total of 396 features was extracted for each patients, including histogram features (42 features), shape factor features (9 features), gray level co-occurrence matrix (GLCM) features (154 features), grey level run-length matrix (GLRLM) features (180 features), and gray level size zone matrix (GLZSM) features (11 features).

### 2.5. Feature Selection and Model Construction

Univarite analysis, spearman correlation analysis and least absolute shrinkage and selection operator (LASSO) is used for dimension reduction of radiomics features. In univariate analysis, the features with *p* < 0.05 were selected. The thresholds for spearman correlation analysis were 0.9. LASSO is the final step of dimension reduction, selecting the most useful predictive features in training set. The radiomics model was constructed by multivariable logistic regression with the selected features. The radscore was obtained by coefficients and intercept item in the radiomics model. The radiomics-clinical model was constructed by radscore and clinical risk factors (*p* value < 0.05 in Table 1).

### 2.6. Model Evaluation

We evaluated the ability of the radiomics feature for differentiation of mycoplasma pneumonia and bacterial pneumonia in the train and test set by the receiver operating characteristic curve (ROC), the area under the curve (AUC) of ROC, sensitivity, specificity, and accuracy.

### 2.7. Nomogram and Decision Curve

A nomogram was constructed based on the radiomics-clinical model. Calibration curves were drawn to evaluate the calibration of the radiomics nomogram. Decision curve analysis was performed to determine the clinical benefit at different threshold probabilities in the validation dataset.

### 2.8. Statistical Analysis

All statistical analyses for the present study were performed with R (version 3.5.1; www.rproject.org, accessed on 13 February 2020) and Python (version 3.5.6; www.python.org, accessed on 13 February 2020). Chi-square test or Fisher’s exact test was used for the nominal variable. Kruskal-Wallis H-test was used for ordinal variable, and t-test was used for continuous variable. The LASSO algorithm is performed using the “glmmet” package in R software. The “rms” package was used to construct the nomogram and corresponding calibration curve. The “rmda” package was used to construct decision curve. ROC analysis was performed using the “pROC” package. A two-tailed *p*-value < 0.05 indicated statistical significance.

## 3. Results

### 3.1. Clinical Characteristic

In the study of 100 patients, clinical data were statistically examined. The results are shown in Table 1, which shows that there were significant differences in age and body temperature between mycoplasma pneumonia and bacterial pneumonia, but no significant differences in gender, c-reactive protein, white blood cell count, and neutrophils count.

### 3.2. Feature Selection and Radiomics Signature Building

**Figure 2:**
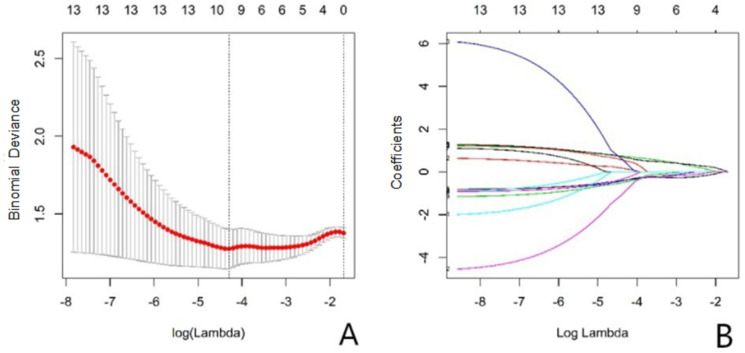
(**A**): Texture feature selection in the LASSO model, displaying the different super parameters (lambda value) that correspond to the diagnostic biases of the different models. The vertical dashed line on the left represents the minimum deviation of log (lambda) at the optimal lambda value, and the dashed line on the right represents the optimal logarithmic value of lambda. The number at the top of the picture is the feature number. (**B**): The variation of LASSO coefficients for different texture parameters as the super parameter (lambda value) changes.

**Figure 3:**
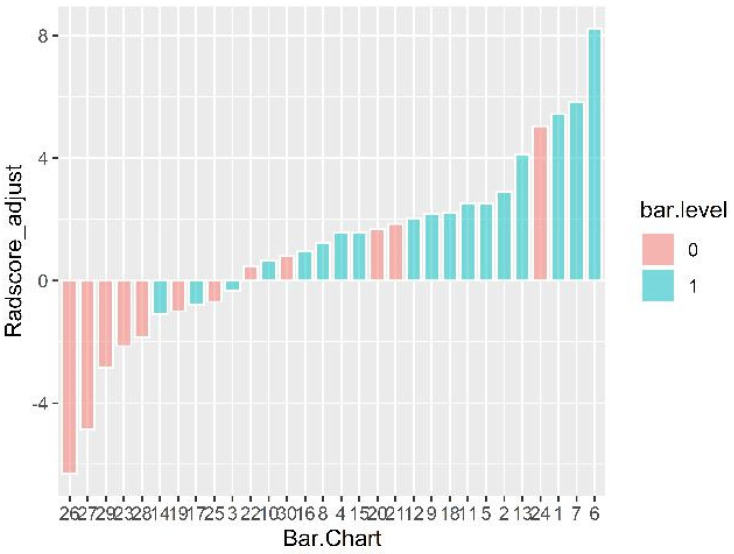
Radscores for each patient are displayed. The horizontal axis represents the serial number of the test set data. Color: pink bar on behalf of bacterial pneumonia; blue bar represents mycoplasma pneumonia. The vertical axis is the radscore value after calibration, namely the original radscore +0.819. Radscore = −0.819 was the generalized cutoff point, if the radscore is higher than the cut-off value, the model would be classified one case into bacterial pneumonia set, otherwise, into mycoplasma pneumonia set.

A total of 396 radiomics features were extracted from the images, and 10 features were selected by univarite analysis, spearman correlation analysis, and LASSO (Figure 2). Figure 3 shows the image score of each patient in the test set.

Radscore is the sum of the features constructed by the model multiplied by the corresponding coefficients, and the formula is “Radscore = intercept +∑βⅰ × Xⅰ”. In this study, Radscore = 0.487 − 1.17 × stdDeviation + 0.74 × Correlation_angle90_offset1 + 2.26 × GLCMEntropy_angle90_offset4 − 1.54 × Inertia_angle0_offset1 − 1.20 × InverseDifferenceMoment_angle45_offset7 + 1.14 × sumEntropy - 1.29 × sumVariance + 1.87 × ShortRunEmphasis_AllDirection_offset7 − 2.70 × ShortRunEmphasis_angle45_offset7 + 2.16 × ShortRunHighGreylevelEmphsaia_angle90_offset7 (Table 2).

### 3.3. Model Evaluation

The ROC curves of the radiomics model and radiomics-clinical model, the diagnostic efficiency of the radiomics model and radiomics-clinical model in train and test set are shown in Table 3. 

### 3.4. Nomogram and Decision Curve

**Figure 4:**
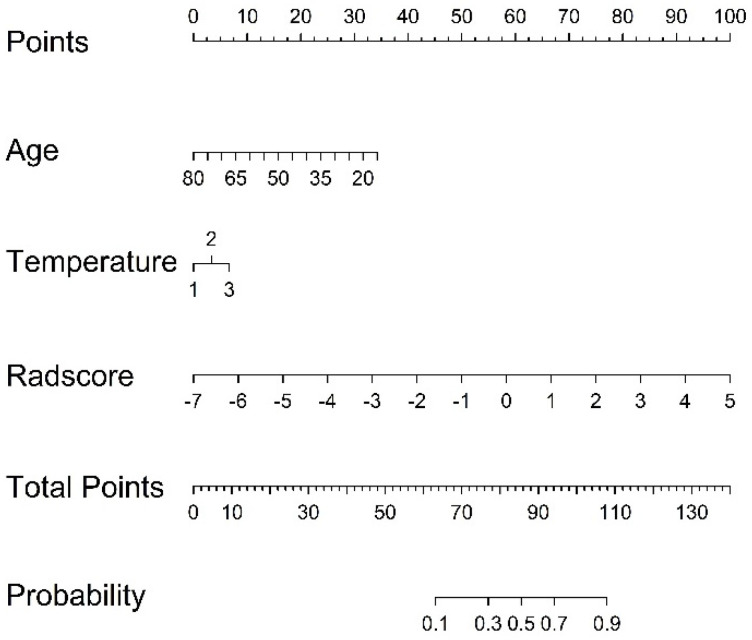
Radiomics nomograms of developed in train set. Note: 1, 2, and 3 in temperature represent low thermal, moderate heat, and high fever, respectively.

**Figure 5:**
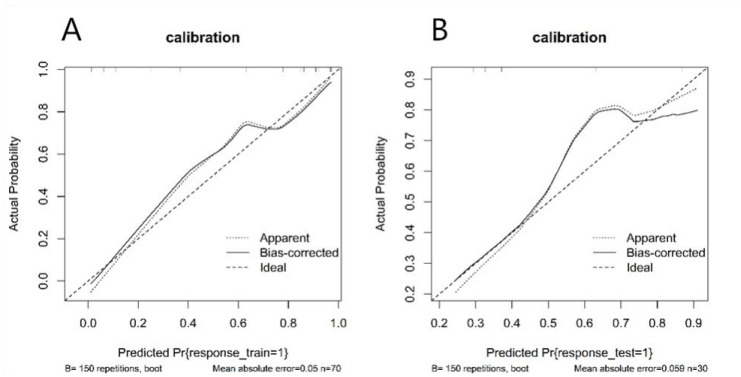
Calibration curve of radiomics nomogram. (**A**): calibration curve of the train set; (**B**): calibration curve of the test set. The *y*-axis shows the actual result. The *x*-axis represents the predicted probability of mycoplasma pneumonia. The diagonal dotted line represents an ideal model. The solid line indicates the performance of the nomogram. If the solid line is closer to the diagonal dotted line, it means a better prediction.

A nomogram was established based on the radiomics-clinical (Figure 4), and the corresponding calibration curve is displayed in Figure 5, which showed the consistency between the predicted classification and actual classification. 

### 3.5. Clinical Application

**Figure 6:**
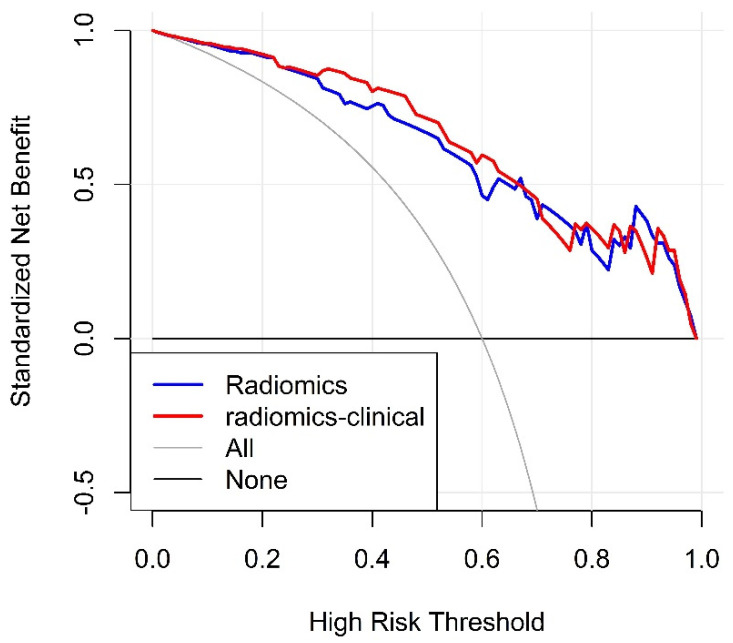
The decision curves. *y*-axis measures the net benefit. The blue dotted line and red dotted line represent the net benefit of radiomics model, radiomics-clinical model, respectively.

The decision curve analysis for the radiomics and radiomics-clinical model is shown in Figure 6. The decision curve shows that radiomics-clinical model gains more clinical benefit over the most threshold range compared to the radiomics model.

## 4. Discussion

In this study, we used chest CT based radiomics to distinguish mycoplasma pneumonia from bacterial pneumonia. To develop radiological features, univarite analysis, spearman correlation analysis, and LASSO logistic regression model was used to reduce the 396 candidate features to 10 potentially highly correlated features. This method is not only superior to the selection method of univariate correlation intensity predictors and results, but can also incorporate selected features into radiological features [12]. More importantly, we found radiomics to be a good differentiator. The sensitivity and specificity of the train set and the test set were 0.762, 0.667, and 0.821, 0.750, AUC was 0.877, and 0.810, and the accuracy rates was 78.6% and 70.0%, respectively. The results of the train set and test set of the comprehensive radiomics model were also relatively satisfactory. Therefore, the radiomics model has the ability to distinguish mycoplasma pneumonia from bacterial pneumonia based on selected features.

Chest CT findings were similar when mycoplasma pneumonia involved alveoli and bacterial pneumonia involved interstitium [13]. The mycoplasma pneumonia lesions generally start from the bronchial mucosal epithelium and appear as edema and thickening of the bronchial wall, with further accumulation of inflammatory cells, which can further develop to the surrounding bronchovascular area and turn into bronchitis and peripheral interstitial inflammation [14,15]. On the HRCT, the central interstitium and bronchial tube wall thickened, and blurred edges and ground glass density foci were observed [16]. The inflammatory lesions continue to develop distally and can continue to be bronchitis, which will lead to narrow bronchial cavity, the formation of intramucosal mucus plugs, the involvement of distal alveoli, and the exudation of alveolar walls and neutrophil plasma cells. On the HRCT, there are tree buds and acinar nodules [16,17]. If the lesion continues to spread toward the surrounding stroma, the affected interstitial lesion appears as a ground-glass density lesion that surrounds and penetrates between the parenchymal lesion and the blood vessels [14,15,18], similar to the fog around the tree, and appears as a tree fog sign on HRCT. As far as bacterial pneumonia is concerned, the direct damage caused by bacteria to the host and the disorder of the body’s immune response are the main factors causing its disease [19]. Some studies have shown that bacteria activate lung epithelial cells and produce inflammatory mediators, causing damage to lung tissue structure and epithelial cells, causing epithelial cell vacuole degeneration and mitochondrial swelling [20]. Intracellular vacuoles collect cytoplasm distortion and cell damage, which further leads to pulmonary endothelial cell apoptosis and alveolar exudation, which is manifested as alveolar consolidation on HRCT [20,21]. When the lesion involves the lung interstitial, it appears as interstitial changes on HRCT, forming peripheral interstitial inflammation.

The damage modes of the inner texture or cells of the two lesions are different, which cannot be distinguished by the naked eye. Radiomics can extract a large amount of information from the images with high throughput, reflecting the heterogeneity within the lesions [22]. This study used 10 radiomics extracted features, where 1 feature belongs to FirstOrderStatistics, 6 features belong to GrayLevelCooccurenceMatrix, and 3 features belong to GrayLevelRunLengthMatrix. StdDeviation is a first-order statistical eigenvalue of voxel strength, which is independent of the distribution of gray intensity in ROI. The other 9 features are higher-order radiomics features that display spatial distribution of pixels. GrayLevelCooccurenceMatrix shows a two-dimensional histogram of pixel grayscale, including Entropy value and the Correlation value. Entropy reflects the intensity of spatial distribution, and the Correlation value reflects the similarity of gray level in adjacent pixels. The higher the entropy value, the higher the lesion heterogeneity [23], indicating that the heterogeneity of inflammatory lesions is greater. This makes it possible for radiomics to distinguish between mycoplasma pneumonia manifesting as peripheral interstitial inflammation and bacterial pneumonia manifesting as alveolar consolidation. In addition, the other features all belong to the GrayLevelRunLengthMatrix texture, which mainly reflects the roughness of texture and directivity. Directional textures will have a longer run at a certain angle, in which the value of short run emphasis on the rougher image is greater, the value of long run emphasis on the smoother image is greater [24], and the lung inflammatory lesions are mainly shown as short run emphasis. The length of the run is related to the distribution of image gray scale, and the heterogeneity of the lesions often reflects the change of image gray scale, so the run matrix is sensitive to the change of pulmonary inflammatory texture. Due to the different mycoplasma pneumonia and bacterial pneumonia pathological changes, although they are visually indistinguishable lesions, different radiomics features can be extracted, which may be the fundamental reason why radiomics can distinguish mycoplasma pneumonia from bacterial pneumonia.

In this study, we collected demographic clinical symptoms, laboratory tests and other relevant factors that may be related to the identification. Statistical analysis was performed for each indicator to select the valuable indicator. The results showed that there was a significant difference in age (*p* < 0.001) and body temperature (*p* < 0.001) between mycoplasma pneumonia and bacterial pneumonia, and no significant difference in gender (*p* *=* 0.165), c-reactive protein (*p* = 0.061), white blood cell count (*p* = 0.126), and neutrophils count (*p* = 0.186). Related studies show that adult mycoplasma pneumonia is more common in young adults, and the results of this study are consistent with the main mycoplasma pneumonia for high fever, bacterial pneumonia for low and moderate heat. Age, temperature index and image score were included in logistic regression analysis to construct a comprehensive model of radiomics and clinical characteristics risk factors to increase the ability of the decision support model [6]. We integrated the radscores and clinical predictive factors to obtain a better comprehensive radiomics model. The sensitivity and specificity of the train set and the test set were 0.976, 0.714 and 0.889, 0.667, the AUC was 0.905, 0.847, and the accuracy was 87.1% and 80.0%. It can be seen that the performance of the integrated radiomics prediction model is significantly better than that of the simple radiomics label, and has some improvements in AUC and sensitivity.

This study has some limitations. First, the nomogram is based on a retrospective analysis, and a prospective study needs to be designed for evaluation and validation. Second, there is also a lack of external validation of the model, and multi-center validation with a larger sample size is needed to obtain high-level evidence for clinical application. We did not classify bacterial pneumonia and further research on the impact of different pneumonia subtypes is necessary. Third, two-dimensional manual segmentation method is adopted to delineate ROI. This method has high accuracy but large individual differences, high time consumption, and low efficiency. Last, radiomics is a discipline that has emerged in recent years. Its research on lung inflammation is still in its infancy. The biological interpretation of the characteristics of radiomics feature need to be explored further in subsequent studies.

In conclusion, this study proposes a radiomics nomogram that combines the characteristics of radiology and clinical risk factors, which can be easily used to identify mycoplasma pneumonia and bacterial pneumonia, so as to provide a basis for early clinical and accurate treatment.

## Figures and Tables

**Table 1:** Clinical Statistics of patients between MP and BP.

Characteristic	MP	BP	*p*
Age, mean ± years	29.68 ± 9.22	43.08 ± 15.94	<0.001 *
Gender, No			0.165
Male	34 (56.67%)	17 (42.50%)	
Female	26 (43.33%)	23 (57.50%)	
Temperature			<0.001 *
Grade 1	7 (11.67%)	17 (42.50%)	
Grade 2	20 (33.33%)	16 (40.00%)	
Grade 3	33 (55.00%)	7 (17.50%)	
C-RP (mg/L)	64.38 ± 49.54	46.89 ± 37.51	0.061
WBC (10^9^/L)	7.69 ± 2.49	8.56 ± 3.10	0.126
Neutrophil (%)	83.06 ± 84.10	65.02 ± 18.62	0.186

Abbreviations: MP, Mycoplasma pneumonia; BP, Bacterial pneumonia; C-RP, C-reactive protein; WBC, White blood cell count; Grade 1, Low thermal (37.1–38.0 °C); Grade 2, Moderate heat (38.1–39.0 °C); Grade 3, High fever (39.1–41.0 °C). * *p* value < 0.05.

**Table 2:** Ten valuable features selected after LASSO.

Radiomics Features	Estimate	Value
stdDeviation	−0.758	−1.17
Correlation_angle90_offset1	0.701	0.74
GLCMEntropy_angle90_offset4	1.154	2.26
Inertia_angle0_offset1	−0.951	−1.54
InverseDifferenceMoment_angle45_offset7	−1.074	−1.20
sumEntropy	1.408	1.14
sumVariance	−1.049	−1.29
ShortRunEmphasis_AllDirection_offset7	2.619	1.87
ShortRunEmphasis_angle45_offset7	−3.371	−2.70
ShortRunHighGreylevelEmphsaia_angle90_offset7	1.219	2.16

**Table 3:** Diagnostic efficiency of the radiomics model and radiomics-clinical model in the train and test set.

Information	Radiomics Model		Radiomics-Clinical Model	
	Train	Test	Train	Test
AUC	0.877	0.810	0.905	0.847
Sensitivity	0.762	0.667	0.976	0.889
Specificity	0.821	0.750	0.714	0.667
Accuracy	78.6%	70.0%	87.1%	80.0%

## Data Availability

No applicant.

## Abbreviation

CAP	Community-acquired pneumonia
HRCT	High-resolution computed tomography
ROI	Region of interest
LASSO	Least absolute shrinkage and selection operator
AUC	Area under the curve
GLCM	Gray level co-occurrence matrix
GLRLM	Gray level run-length matrix
GLZSM	Gray level size zone matrix
MP	Mycoplasma pneumonia
BP	Bacterial pneumonia
C-RP	C-reactive protein
WBC	White blood cell count
